# How Co-translational Folding of Multi-domain Protein Is Affected by Elongation Schedule: Molecular Simulations

**DOI:** 10.1371/journal.pcbi.1004356

**Published:** 2015-07-09

**Authors:** Tomohiro Tanaka, Naoto Hori, Shoji Takada

**Affiliations:** Department of Biophysics, Graduate School of Science, Kyoto University, Kyoto, Japan; University of Maryland, UNITED STATES

## Abstract

Co-translational folding (CTF) facilitates correct folding *in vivo*, but its precise mechanism remains elusive. For the CTF of a three-domain protein SufI, it was reported that the translational attenuation is obligatory to acquire the functional state. Here, to gain structural insights on the underlying mechanisms, we performed comparative molecular simulations of SufI that mimic CTF as well as refolding schemes. A CTF scheme that relied on a codon-based prediction of translational rates exhibited folding probability markedly higher than that by the refolding scheme. When the CTF schedule is speeded up, the success rate dropped. These agree with experiments. Structural investigation clarified that misfolding of the middle domain was much more frequent in the refolding scheme than that in the codon-based CTF scheme. The middle domain is less stable and can fold via interactions with the folded N-terminal domain. Folding pathway networks showed the codon-based CTF gives narrower pathways to the native state than the refolding scheme.

## Author Summary

Proteins are synthesized *in vivo* by ribosome from their N-termini. When N-terminal fragments of nascent proteins get out of the ribosome exit, they start folding, which is called co-translational folding. It has been suggested that well-scheduled co-translational folding schemes would facilitate correct acquisition of their native structures for some multi-domain proteins. In particular, an un-ambiguous experiment was recently reported for a model protein, SufI where pauses at certain positions in the translational elongation are obligatory for efficient folding. Here, for the first time to our knowledge, we performed molecular dynamics simulations of SufI with co-translational folding as well as re-folding schemes. We found a co-translational folding shceme with rare codon-based pauses indeed increased the success ratio of folding, which is consistent with recent experiments. On top, molecular simulations provided much of structural insights on the folding routes and misfolding in the case of re-folding scheme. This explains why pauses in the translational elongation rescue SufI from misfolding.

## Introduction

While *in vitro* folding dynamics of single-domain proteins has been relatively well understood by now[1,2], several additional factors make *in vivo* protein folding much more difficult to characterize. About 70% of proteins have multiple domains and inter-domain interactions often cause many metastable intermediates and can hamper folding to the native states [3,4]. Cellular environment is highly crowded by macromolecules, which affects folding kinetics and could cause aggregation [5–7]. To circumvent some of these difficulties, several types of molecular chaperones facilitate folding [8]. During protein synthesis in ribosome, nascent polypeptides start folding co-translationally [9].

Co-translational folding (CTF) has been suggested for *in vivo* folding mechanism since 1960’s [10] and there is no room to doubt its relevance both in bacteria and in eukaryotic cells [11]. Many elements in the CTF have been characterized [12]. First of all, many proteins, once denatured in a test tube, do not refold with high probability, whereas they fold in the CTF condition. Thus, as a rule of thumb, the CTF condition facilitates correct folding of many proteins [13,14]. Ribosome is not just a machine for synthesis, but also helps folding of nascent chains at the exit tunnel and on the surface [15]. The translation elongation is not at uniform rate, but there are some regions on mRNA where the elongation is markedly slowed down [16–18].

This so-called elongation attenuation can be realized by a few mechanisms. Most notably, for a given codon, the elongation rate is affected by its cognate tRNA binding kinetics, thus depending on the concentration of the cognate tRNA [19]. The concentration of cognate tRNAs are highly correlated with the frequencies of codon usage for each of species. There are some codons, of which the cognate tRNAs have markedly low concentration[19]. These rare codons sometime appeared in mRNA as a cluster, which often leads to translational attenuation. On top, some portions of mRNA form partial secondary structures, which may slow down the elongation contributing to the elongation attenuation as well[20]. It was anticipated that the locations of the attenuation might have evolved to facilitate the CTF. Some of them appear near domain boundaries of multi-domain proteins [21]. By synonymous substitution of rare codons, one can speed up the translation elongation at a certain position without changing amino acid sequence, which led to reduce or impair functions and/or protease resistance for some proteins, such as an acetyl-transferase [22] and SufI [16].

**Fig 1:**
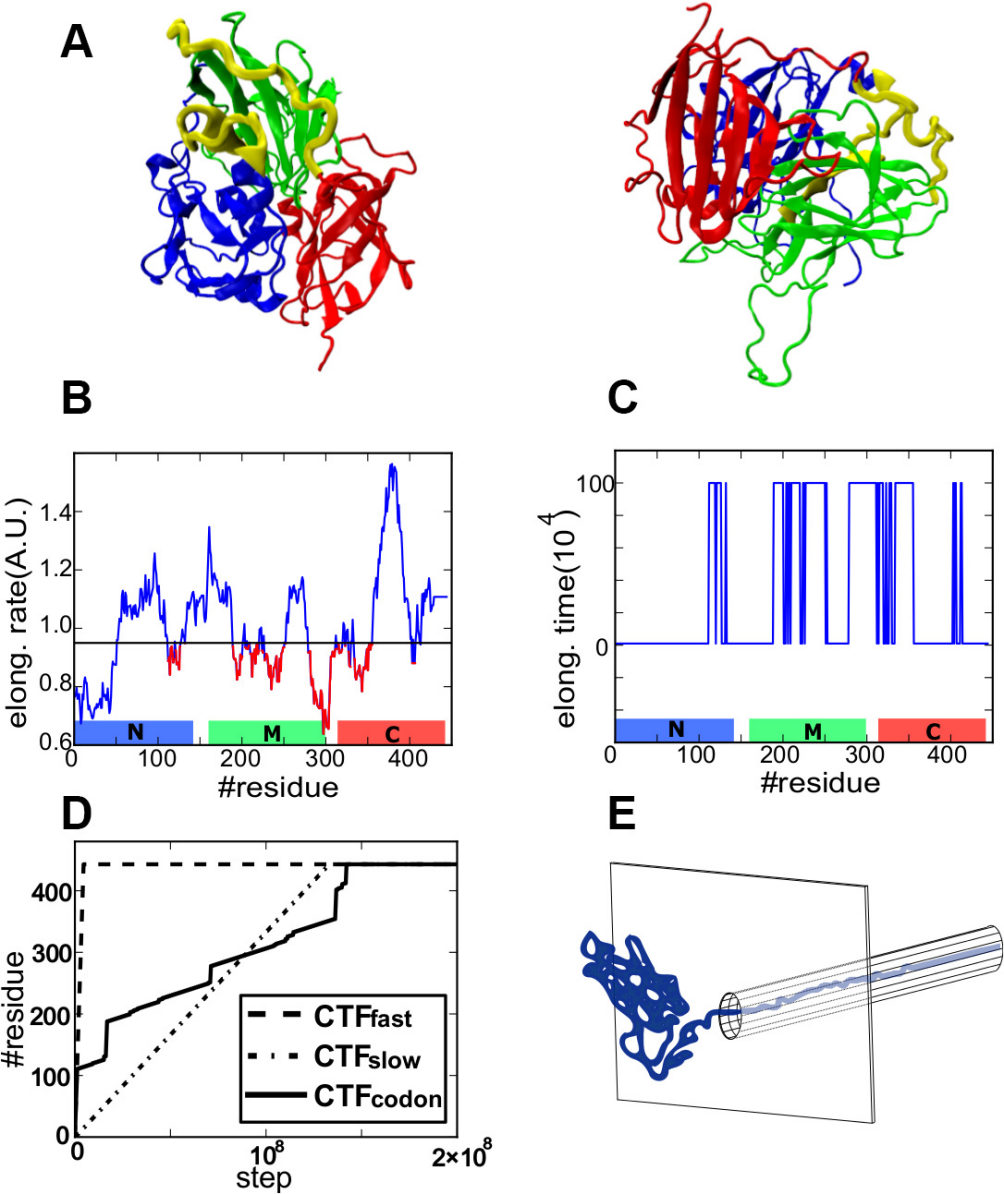
Simulation setup. A) Strucuture of SufI. N-, M-, and C-domains are depicted in blue, green, and red. Linkers that connet two domains are depicted in yellow. B) The codon-based elongation rate by Spencer *et al*’s algorithm. A threshold is introduced. The region where the elongation rate is slower than the threshold is drawn in red. C) The elongation schedule used in the CTF_codon_ simulations. Regions marked in red in B take long elongation time. D) Three CTF schems. The CTF_fast_ (dashed), the CTF_slow_ (dotted), and the CTF_codon_ (solid) lines. E) A schematic view of the system including the wall-and-tunnel potential.

SufI in *E*. *coli* was recently used to test the role of translational attenuation in the CTF [16]. SufI, an about 450-residue protein, is made of three domains; N- (blue in Fig 1A), M- (green), and C- domains (red), in order. Zhang *et al*. first identified three clusters of rare codons, two of which indeed exhibited elongation attenuation [16]. Synonymous substitutions of some rare codons in these regions led to reduction or impair of protease resistance. Separately, using a cell-free system, they also increased the concentrations of the corresponding tRNAs, which showed the similar results to the above synonymous substitution experiment. It should also be noted that, they found no interactions of SufI with molecular chaperones. Thus, these experiments provide us an unambiguous evidence of biological importance of the elongation attenuation for efficient folding in the CTF condition.

These experimental data can be complemented with theoretical and computational analysis to deepen our understanding on the CTF mechanisms. Previously, lattice Monte Carlo simulations [23,24] and statistical theories [25,26] addressed physical aspects of CTF mechanisms. Coarse-grained molecular dynamics (CG MD) was used to investigate interaction with ribosome in the CTF [15,27–29]. These works helped understanding general and conceptual aspects of the CTF, but they were not specific enough to compare with experimental data of specific substrate proteins. It is time to start computational study of CTF for a specific protein, of which clear experimental data are available. This enables us to address structural aspects of CTF mechanisms, which is indeed the purpose of this work and we chose SufI for it.

Since the CTF becomes non-trivial primarily for relatively large and multi-domain proteins (SufI has three domains and is about 450 residue long (Fig 1A)), all-atom MD simulations are not feasible for this problem at the moment. By now, no all-atom MD simulation for folding to the native structure of multi-domain proteins was reported. To overcome size and time scale limit in all-atom MD simulations, protein folding simulations have commonly performed by coarse-grained (CG) models that are based on the energy landscape theory [30,31]. In particular, these simulations include medium-to-large proteins, such as multi-domain proteins[32–34].

Yet, to address mechanisms of the CTF and, in particular, an impact of elongation attenuation by CGMD simulations, technically, there are two major issues. First, we need to realize misfolding as well as correct folding in a well-balanced manner. Thus, the CG model needs to be calibrated so that an energy landscape is globally funneled in one hand and modestly rugged in the other hand. There have been a considerable number of studies towards hybrid modeling of structure-based potentials for globally funneled landscape with sequence-dependent terms for modestly rugged surfaces [35]. Yet, it should be noted that, currently, there is no established manner to balance the two aspects. Thus, here we unavoidably take a heuristic and empirical approach. Second, we need to design a scheme that mimics co-translational folding *in silico*. Quantitative kinetic measurements and detailed mechanisms of translation attenuation are not available at the moment, which led us to take a rather simplistic modeling of CTF scheme. Albeit these limitations, with the current CGMD, we can simulate complete folding and misfolding events of full-length SufI hundreds of times in scheme that mimics the CTF.

In this paper, we first describe computational modeling of CGMD for the CTF. Then, we performed the CTF and, as a control, the refolding simulations of SufI, comparing these results. Characteristics of misfolded structures are then analyzed. Next, folding networks for these simulations clarify impacts of CTF and the elongation attenuation on folding reaction mechanisms. Finally, the correlation between the degree of folding and the translation elongation time was investigated.

## Results and Discussions

### Computational modeling

In the current CG modeling, each amino acid is represented by one bead located at the C_α_ position. For folding simulations by CGMD, the so-called perfect-funnel model, or often called Go model, has been widely used giving many insightful lessons for folding dynamics [36–39]. However, the perfect-funnel approximation may not be sufficient to study CTF dynamics where successful folding competes with misfolding, or non-native traps. The latters are, by definition, not realized by the perfect funnel approximation. To this end, here we developed a hybrid CG model where we added a generic hydrophobic (HP) interaction potential *V*_*HP*_ to the Go model potential *V*_*Go*_; the latter is responsible for globally funnel-like shape of the landscape, while the former makes the landscape modestly rugged leading to many metastable non-native traps. Concretely, the entire potential function of a protein is *aV*_*Go*_+*bV*_*HP*_. The Go potential was parameterized based on the atomic interaction at the native structure, called the AICG model developed by Li *et al* [32]. The HP interaction is a generic many-body potential that estimates how a hydrophobic residue is buried by other residues [40]. The HP interactions were applied not only natively interacting pairs, but also any residues. Detailed potential functions are described in Materials and Methods, Coarse-grained model.

As is well-known, proteins *in vivo* are gradually synthesized by ribosome from their N-termini and released from the ribosome exit tunnel, which we try to mimic in a simple manner. In the protocol, amino acids are added one by one to the C-terminus of the nascent polypeptide chain with certain “translation” rates (Fig 1B and 1C. See Materials and Methods, Coarse-grained model for details). To investigate effects of elongation attenuation, we employed the following three translation rate schemes (Fig 1D): 1) The uniformly fast translation scheme (a dashed line, designated as CTF_fast_), 2) the uniformly slow translation scheme (a dotted line, CTF_slow_), and 3) the non-uniform codon-based translation scheme (a solid line in Fig 1D and 1C, CTF_codon_) that is dependent on the cognate tRNA concentration. We note that, in our scheme, the *in silico* translation rate is not proportional to the translation rate predicted from cognate tRNA concentrations. The translation attenuation was linked to a cluster of rare codons, which implies that the attenuation is a collective phenomenon and possesses distinct phases. Thus, using a threshold of the predicted translation rate, we introduced a two-phase approximation where the *in silico* translation is either "normal" or "slowed". The slowed translation phase, of which translation speed is 100 times slower than the normal case, corresponds to the translation attenuation. Since there is no quantitative kinetic measurement on the attenuation, this two-phase approximation and use of slowing factor 100 are rather simple, possibly over-simplified, schemes. Yet, we consider it qualitatively captures some of the major features of the translation attenuation. As far as the slowed phase is sufficiently slower that the normal phase, we expect qualitatively similar results. The relation between the translational time scales and the inherent folding time scales is of crucial importance, which will be discussed at the end of the results. Detailed *in silico* elongation scheme is described in Materials and Methods, Translational elongation scheme. Additionally, *V*_*tunnel*_ was introduced to mimic the ribosome steric effect that is realized by a combination of a wall and a tunnel (Fig 1E). Note that we did not include any molecular representation of ribosome and thus the tunnel is merely to restrict the nascent chain in a confined geometry. During elongation, a polypeptide chain is tethered to the base of the tunnel. On average, about 28 residues resided in the tunnel (S1 Fig). After completing the elongation, the chain is released from the base. We note that the exit tunnel was included to account for the gap between the residue at the catalytic center and the segment that can fold. The codon-based translation rate is based on the codon (sequence) at the catalytic center. In principle, some alpha helical structures can be formed in the exit tunnel depending of the sequence (although, retrospectively, we did not find it).

For comparison, we also performed folding simulations of SufI in a refolding scheme, where a full-length polypeptide chain started folding from denatured conformations obtained by high temperature simulations. No wall-and-tunnel potential *V*_*tunnel*_ was utilized in this scheme.

MD simulations were performed at 0.82*T*_*F*_ *, where *T*_F_ * is an upper limit of denaturation temperature in our CG model. To determine the temperature, starting from the native state of SufI, we performed unfolding simulations for 1 x 10^8^ time steps at many temperatures. The lowest temperature at which we observed unfolding was defined as the upper limit of denaturation temperature *T*_F_ *. (S2 Fig). We note that, even with the CG modeling, accurately calculating the denaturation temperature is a formidable task for this size of proteins; using the standard replica-exchange method or multi-canonical ensemble method, we did not succeeded to obtain the reversible folding/unfolding trajectories.

### Co-translational folding and refolding simulations

**Fig 2:**
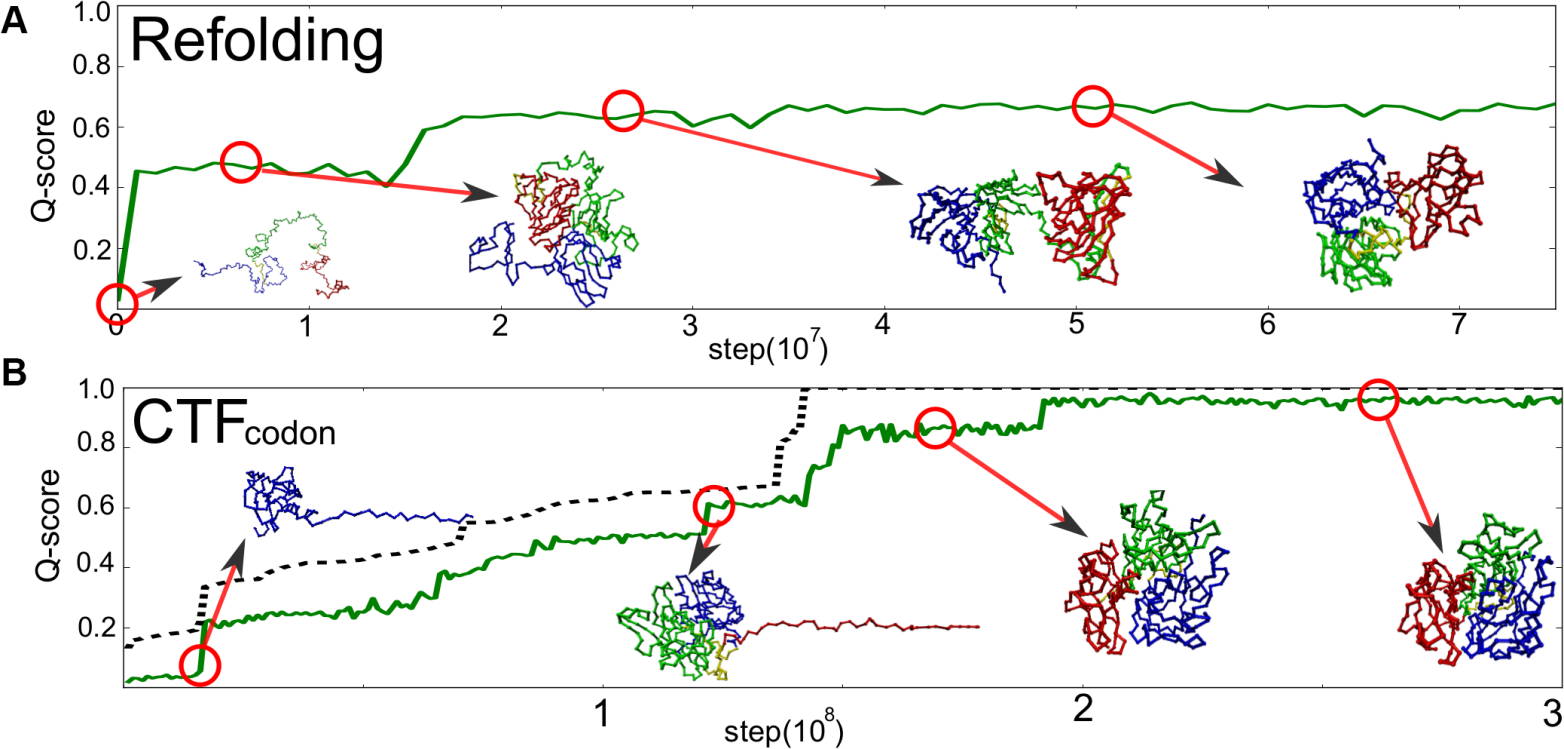
Representative time courses of folding simulations. A) A time course of a refolding trajectory. B) that of the CTF_codon_. Some snapshots were drawn with the same color code as Fig 1A.

First we compare a representative folding trajectory via the codon-based co-translational folding (CTF_codon_) scheme with that via the refolding scheme. Fig 2 illustrates folding time courses quantified as the so-called Q-score defined as the fraction of formed contacts that exist at the native structure, together with some representative snapshots.

In the refolding trajectory shown in Fig 2A, the protein first acquired one globular region, which roughly corresponds to the N-domain. After a while, another globular region was formed, which contains, roughly, the C-domain and a half of M-domain. They gradually coalesced and made a single globular structure, which was a deep misfolded trap; the protein stayed in this trap until the end of the simulation.

On the other hand, the CTF_codon_ trajectory in Fig 2B showed markedly different time course. A cooperative folding of the N-domain at ~ 0.2 × 10^8^ time step is followed by the folding of M-domain at ~ 1.2 × 10^8^ time step. Subsequently, at ~ 1.7 × 10^8^ time step, the protein folded to near native structure in which the C-domain is partly misfolded. Finally, at around 1.9 × 10^8^ time step, it quickly transited into the native-like conformation.

More quantitatively, we repeated folding simulations of SufI 100 times both in the refolding and the CTF_codon_ schemes. In each trajectory, we judged whether the protein is folded or not by a set of native-ness scores, Q-scores, at the final 100 structures of the simulation (0 ≤ *Q* ≤ 1. Q = 1 at the native structure. We have both a generous and a stringent criteria for the judgment of folding. See Materials and Methods, Criteria for folding for more detail). Using a stringent criterion of folding, of 100 trajectories we found 18 successful folding cases in the refolding scheme (Table 1). Whereas, the CTF_codon_ resulted in 35 cases of correct folding. To clarify the statistical significance of the difference, we computed the histograms of Q-scores of the final structures in each scheme (S3 Fig). The difference in Q-score probability distributions was tested by the Kolmogorov-Smirnov test, which gave p-value of 0.000174 (Table 2, See also Table 3 for pairwise Mann-Whitney U tests). Thus, we conclude that the codon-based CTF simulation can fold SufI with significantly higher probability than the refolding can do.

**Table 1:** Number of successful folding cases^a^ out of 100 trajectories.

scheme	Refolding	CTF_fast_	CTF_slow_	CTF_codon_
folded	18	20	25	35

^a^ For the judgement of folding, the stringent criterion was used.

**Table 2:** Pairwise Kolmogorov-Smirnov tests to check the difference of the histograms of Q_total_-scores of the final structures.

scheme	Refolding	CTF_fast_	CTF_slow_	CTF_codon_
Refolding	1.0	0.556	0.0314	0.000174
CTF_fast_		1.0	0.140	0.00822
CTF_slow_			1.0	0.556
CTF_codon_				1.0

**Table 3:** Pairwise Mann-Whitney U tests to check the difference of the histograms of Q_total_-scores of the final structures.

scheme	Refolding	CTF_fast_	CTF_slow_	CTF_codon_
Refolding	1.0	0.354	0.00838	0.000189
CTF_fast_		1.0	0.106	0.00779
CTF_slow_			1.0	0.305
CTF_codon_				1.0

### Effects of translational attenuations in co-translational folding

We then investigate effects of translational attenuation regions in SufI sequence, that was studied in experiments [16]. Experimentally, accelerating translation at certain slow translating regions, either by synonimous substitutions or by increasing concentrations of the rare tRNAs, inpaired SufI functions, most likely, due to misfolding. To test this idea in simulations, we conducted folding simulations by the CTF scheme, in which the chain is elongated with a uniform and fast rate across the entire chain (CTF_fast_).

In the same way as the CTF_codon_ case, we repeated the CTF_fast_ simulations 100 times. Using the same criteria for the judgment of folding, i.e., Q-scores, we found only 20 cases of successful folding, which is much fewer than the CTF_codon_ scheme. The statistical analysis of the distribution suggested that the difference is significant (p = 0.00822). Actually, the result by the CTF_fast_ scheme is statistically indistinguishable to that by the refolding scheme (p = 0.556). This is consistent with the experiment of Zhang *et al* [16].

Experimentally, lowering temperature could rescue the low-folding yield of the impaired folding scheme, which we now test in simulations. For the purpose, we performed folding simulations of SufI by the CTF where the elongation is slow and is in a uniform rate entirely (CTF_slow_). Of 100 simulations, we found 25 successful foldings by the same criteria as above. The statistics test resulted in no significance between th CTF_slow_ and the CTF_codon_ schemes, while a subtle p value, p = 0.14 for the comparison between the slow and the fast CTF schemes.

To understand the CTF, comparison between the translation time scale and the folding time scale is of central importance. To estimate relevant folding time scales, for individual domains, we performed kinetic folding simulations. Time required to reach structures that have Q > 0.5 was computed for each of domains (S4 Fig). First, the M-domain is rather unstable and we could not observe successful folding of the standalone M-domain. The time scale for rough folding of N-domain *τ*_*N*−*fold*_ was 1.4 × 10^7^ time steps, which is longer than that of the C-domain, *τ*_*C*−*fold*_ = 3.6 x 10^6^ time steps. Interestingly, *τ*_*N*−*fold*_ is longer than the time to complete translation by the CTF_fast_ scheme, *τ*_*translation*−*fast*_ ~ 4.4 × 10^6^, but is comparable to that by the CTF_slow_ scheme, *τ*_*translation*−*slow*_ ~ 1.3×10^7^. Importantly, when the time for completion of the translation of N-domain is comparable to or longer than the average folding time of N-domain, the success ratio of SufI is high.

### Misfolded structures

**Fig 3:**
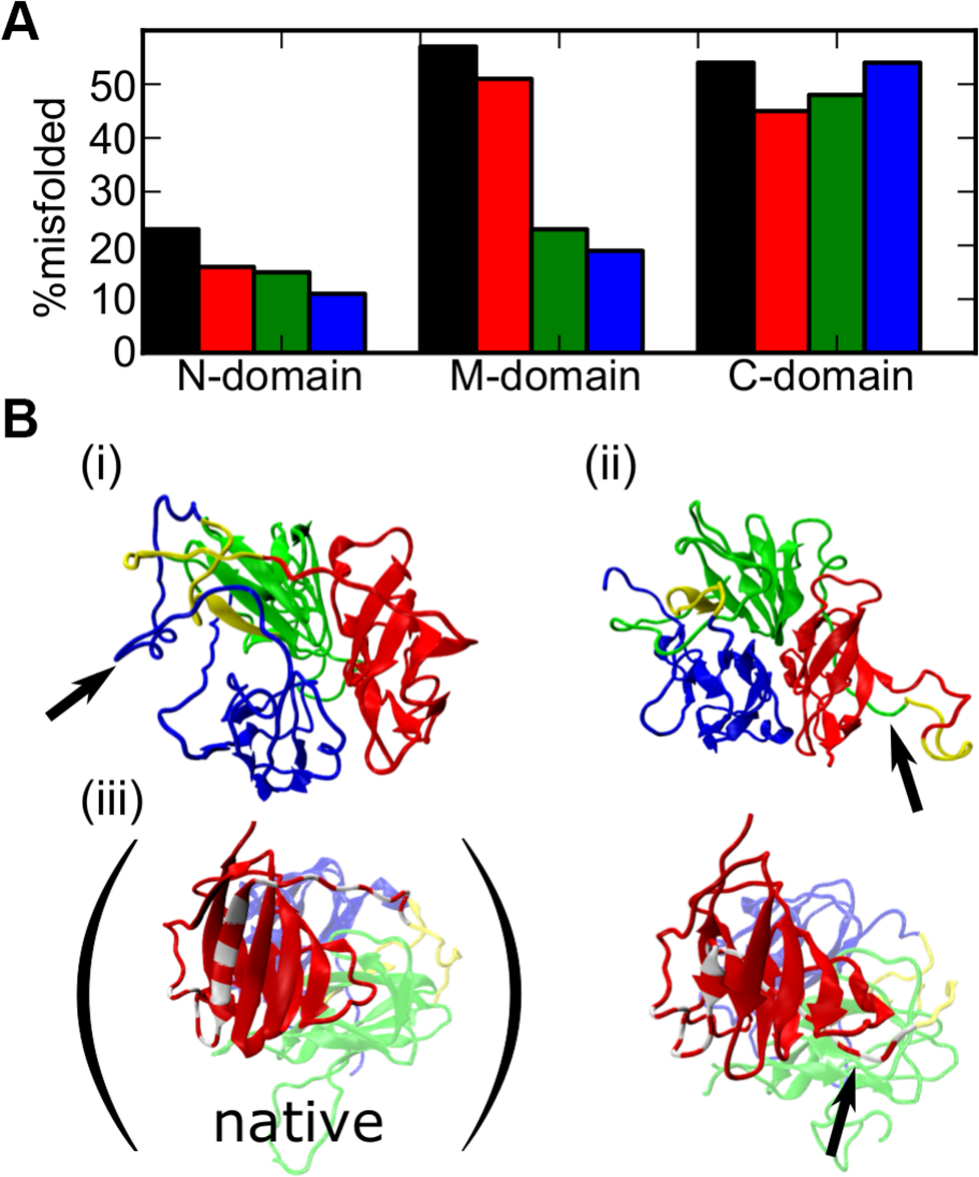
Misfolding in SufI domains. A) Fractions of misfolded domains at the end of simulations in four different schemes; the refolding (black), the CTF_fast_ (red), the CTF_slow_ (green), and the CTF_codon_ (blue). B) Representative final structures of misfolding. i) structure that is misfolded in N-domain. ii) Misfolded in M-domain. iii) (right) Misfolded in C-domain. (left) Native structure for comparison. See text for the explanation of the block arrows.

To understand why the codon-based CTF can facilitate folding of SufI, we now look into misfolded structures. For each of the four folding schemes, we analyzed probabilities of misfolding of individual domains at the ends of simulations (Fig 3A. Statistical test given in Tables 4–9). Here, the misfolded state was judged by the Q-scores of individual domains (To help understanding of typical Q-scores in SufI, we tabulated Q-scores of individual domains as well as those of interface for every snapshots in Fig 2 as S1 Table). Clearly, misfolding in the N-domain and the M-domain occurred with the highest probability by the refolding scheme, which is followed by the CTF_fast_ scheme. The CTF_codon_ showed the smallest probabilities of misfolding for these domains. Of the four schemes, the rank order in misfolding of N- and M-domains is well (anti-)correlated with the probability of successful folding of the full-length SufI. (Table 1) In particular, probabilities of misfolding of the M-domain are markedly different between the refolding and the codon-based CTF. We note that the M-domain is not very stable and cannot fold as an isolated domain (S4 Fig). Folding of M-domain is achieved by structural support of the N-domain. In CTF schemes, when M-domain is synthesized and released from the exit tunnel, the N-domain has large chance to be folded. The folding of the C-domain is not much different among the four schemes.

**Table 4:** Pairwise Kolmogorov-Smirnov tests to check the difference of the histograms of N-domain’s Q-scores of the final structures.

scheme	Refolding	CTF_fast_	CTF_slow_	CTF_codon_
Refolding	1.0	0.0994	0.261	0.0470
CTF_fast_		1.0	1.0	0.794
CTF_slow_			1.0	0.556
CTF_codon_				1.0

**Table 5:** Pairwise Kolmogorov-Smirnov tests to check the difference of the histograms of M-domain’s Q-scores of the final structures.

scheme	Refolding	CTF_fast_	CTF_slow_	CTF_codon_
Refolding	1.0	0.677	5.96E-6	5.22E-8
CTF_fast_		1.0	0.000581	2.85E-6
CTF_slow_			1.0	0.261
CTF_codon_				1.0

**Table 6:** Pairwise Kolmogorov-Smirnov tests to check the difference of the histograms of C-domain’s Q-scores of the final structures.

scheme	Refolding	CTF_fast_	CTF_slow_	CTF_codon_
Refolding	1.0	0.677	0.261	0.0691
CTF_fast_		1.0	0.261	0.0131
CTF_slow_			1.0	0.677
CTF_codon_				1.0

**Table 7:** Pairwise Mann-Whitney U tests tests to check the difference of the histograms of N-domain’s Q-scores of the final structures.

scheme	Refolding	CTF_fast_	CTF_slow_	CTF_codon_
Refolding	1.0	0.0265	0.0406	0.00218
CTF_fast_		1.0	0.886	0.433
CTF_slow_			1.0	0.322
CTF_codon_				1.0

**Table 8:** Pairwise Mann-Whitney U tests to check the difference of the histograms of M-domain’s Q-scores of the final structures.

scheme	Refolding	CTF_fast_	CTF_slow_	CTF_codon_
Refolding	1.0	0.509	4.89E-5	3.69E-7
CTF_fast_		1.0	0.000594	9.98E-6
CTF_slow_			1.0	0.577
CTF_codon_				1.0

**Table 9:** Pairwise Mann-Whitney U tests to check the difference of the histograms of C-domain’s Q-scores of the final structures.

scheme	Refolding	CTF_fast_	CTF_slow_	CTF_codon_
Refolding	1.0	0.434	0.575	0.360
CTF_fast_		1.0	0.266	0.189
CTF_slow_			1.0	0.807
CTF_codon_				1.0

We now show some representative misfolded structures (Fig 3B). A conformation in Fig 3B (i) taken from a refolding trajectory, is misfolded in the N-domain, while the M- and C-domains are well-folded (The non-native contact map is given in S6 Fig (i)). In this structure, C-terminal end of the N-domain is unfolded and is flipped out to the left side in the figure (See the block arrow. Also, see S6 Fig (i) for many non-native contacts in C-terminus of the N-domain). With this flipped-out segment, three domains coalesced to form near-native domain-domain interfaces. Once the interfaces are firmly formed, the protein is topologically trapped and an escape event from this trap was not realized. Fig 3B(ii) illustrates a case where C-terminus segment of the M-domain was entangled with the C-domain (the block arrow. See also the non-native contact map S6 Fig (ii)). Again, the domain-domain interfaces are near-native like, which makes an escape from this trap difficult. The right cartoon of Fig 3B(iii) shows the case that a N-terminal segment of the C-domain, 314–340 residues (shown in red-and-gray striped pattern with block arrow) goes through different paths from the native structure (the left cartoon of Fig 3B (iii)).

### Folding network

Next, we investigated the ensemble of folding pathways for the CTF and the refolding schemes. To clarify folding pathways, we drew folding networks where nodes represent discretized conformational states and links represent transitions between the states[41,42].

Conformational states were discretized by the native-ness scores (Q-scores) and by the non-native contact scores (N-scores) (See Materials and Methods, Discretization of states by Q-scores of parts for more details). For each domain and each interface between domains, we defined Q-score and N-score (we have six Q- and N-scores, in total). As usual, the Q-score measures fraction of formed native contacts. The N-score is defined as the number of non-native contacts normalized by its maximal number observed. Each Q-score is categorized into 5 classes, while each N-score is divided into 3 classes. Together, we have as many as 5^6^ × 3^6^ ~ 1.1 × 10^7^ states (nodes). To simplify the network, we removed any loops that go from a node and return to the same node later. All 100 trajectories were used to draw a network for each folding scheme.

**Fig 4:**
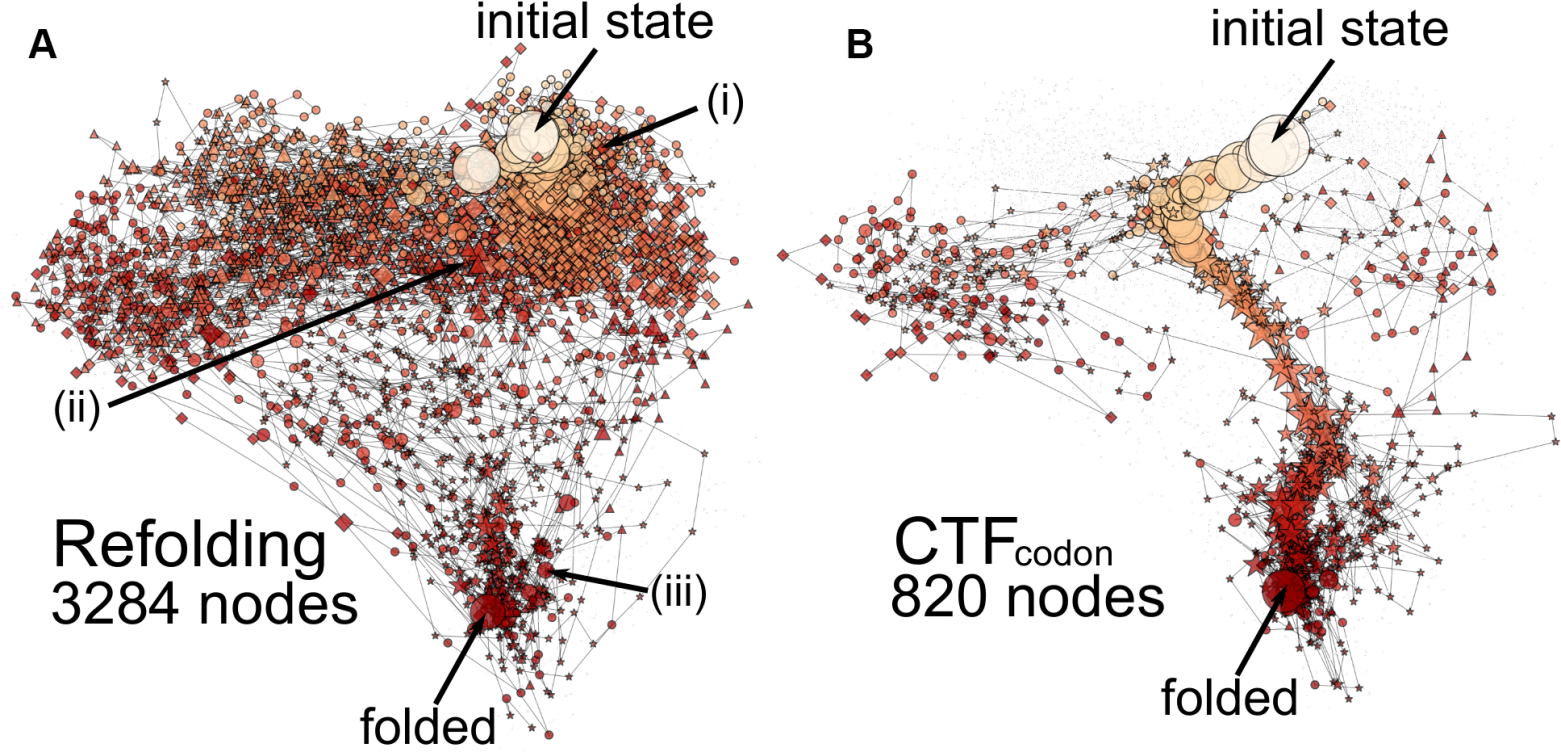
SufI folding networks for refolding (A) and for the codon-based CTF (B). The refolding network possesses 3284 nodes, while the codon-based CTF has only 820 nodes. The size of nodes represent their probabilities. The darkness of the node represents native-ness. The darker one is closer to the native. Diamonds, triangle, and stars indicate that N-, M-, and C-domains are pre-dominantly unfolded, respectively. When pre-dominantly unfolded domains are no uniquely decided, circles are used.

We depict folding networks of SufI for four different folding schemes (Figs 4 and S5). Comparing the folding networks of the refolding (Fig 4A) and the CTF_codon_ (Fig 4B) schemes, we found, first of all, that the network for the refolding has much more nodes (3284 nodes) than the CTF_codon_ has (820 nodes). By refolding, the protein exhibited much more divergent conformational states, many of which are characterized by low Q-scores and high N-scores. Second, while the refolding scheme did not show any dominant pathways, the CTF_codon_ has a clear folding route from the top in the figure to the bottom. Obviously, the CTF enforced SufI to fold vectorially from N-terminal, which provided constraints to the order of domain folding events. In contrast, the refolding scheme made a protein fold freely from any segments resulting into diverse transitions. The CTF restricts kinetics of proteins and reduces conformational ensemble being observed, and are consistent with earlier theoretical works[23].

The folding network for the CTF_fast_ scheme (S5A Fig) apparently looks similar to that of the refolding. The number of nodes found was 3108, which is only slightly fewer than that of the refolding network, i.e. 3284. The transitions are diverse with no dominant pathway to the native state.

On the other hand, the slow CTF scheme showed the folding network (S5B Fig) rather similar to that in the CTF_codon_ scheme. The number of nodes found in the slow CTF was 1096, which is slightly larger than that found in the CTF_codon_, i.e. 820. We see a single and nearly identical folding route in these two schemes.

### Correlation between translation rate and folding

**Fig 5:**
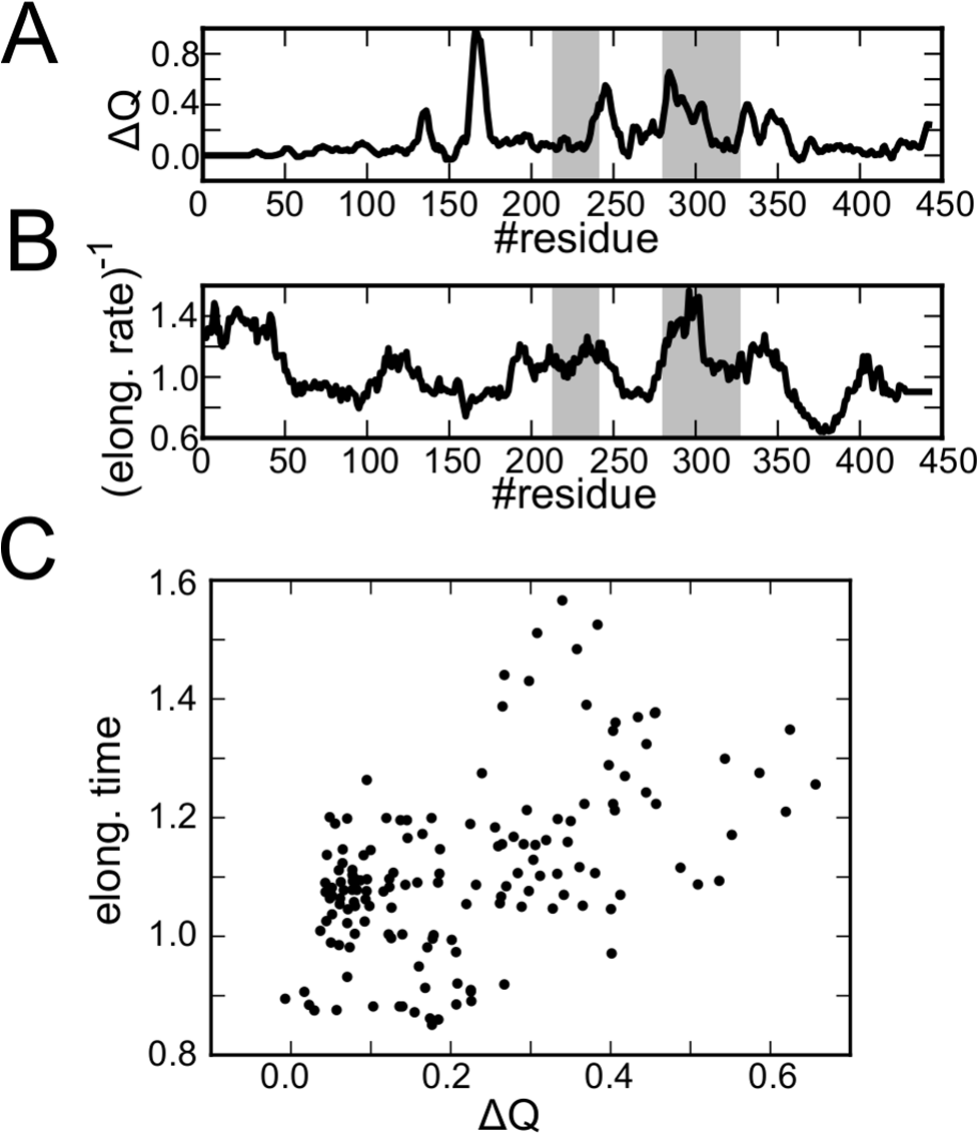
The correlation between the inverse of elongation rate and the degree of folding in SufI. A) The degree of folding acquisition Δ*Q*_*i*_ after averaging over the window size 5. B) One over the translation rate computed from the Spencer *et al*.’s algorithm [43]. Experimentally-detected translational attenuation regions, 33-40kDa (281-326th residues) and 25-28kDa (214-240th residues), are shaded in grey [16]. C) The scattered plot of the translation time and the degree of folding. Here, residues 200–350 are used. The correlation coefficient was 0.51.

It is interesting to ask to what extent the translation rate is designed (optimized), via codon usage, to facilitate folding. To this end, here we investigate the correlation, if any, between a putative translation rate and the degree of folding. For the former, we simply use the translation rate, in arbitrary unit, predicted by an algorithm proposed in Spencer *et al* (Fig 5B) [43]. This translation rate is encoded in the codon usage as well as tRNA concentrations and other factors, but not apparently dependent on the physical chemistry of folding. For the degree of folding acquisition, we defined the progress of native-ness Δ*Q*_*i*_ in a nascent chain of the length *i* as

$$\Delta Q_{i}={\langle {\langle Q\rangle }_{L=i}-{\langle Q\rangle }_{L=i-1}\rangle }_{100\;trajectories}$$

where 〈*Q*〉_*L*=*i*_ is the average *Q*-score when the nascent chain has the length *i* and 〈 〉_100 *trajectories*_ means the average over 100 trajectories in the slow CTF scheme. If Δ*Q*_*i*_ is high at *i*-th residue, a nascent chain gains Q-score without disturbance from more C-terminal region of the chain. Note that if we used the codon-based CTF scheme in calculation of Δ*Q*_*i*_, it would naturally correlate with the translation rate. Importantly, however, we did not bias the CTF by the codon usage. Instead, we used a uniform and slow CTF scheme. Thus, Δ*Q*_*i*_ is not directly related to the difference in the translation rate, but is a purely physicochemical quantity determined by the amino acid sequence. We note that Δ*Q*_*i*_ was smoothed by a window average of the 5-residue windows to reduce the noise.

The Δ*Q*_*i*_ profile shown in Fig 5A exhibits several peaks. First, we focus on the peaks that correspond to folding of M-domain because it is the most difficult event. We find a high Δ*Q*_*i*_ region around 280–310, which well correlates with a translational attenuation region, 33-40kDa region (281–326 residues, grey shaded in Fig 5). Experimentally, synonymous substitutions of rare codons in this region reduced resistance to a protease [16]. The other translational attenuation experimentally tested is 25-28kDa (214–240 residues), in which synonymous substitution of two leucine codons impaired the protease resistance of SufI. In Fig 5A, we see peak in the Δ*Q*_*i*_ profile at ~245. More quantitatively, by using 200th-350th residues, we computed the correlation between the Δ*Q*_*i*_ profile and the translation rate profile (Fig 5C) finding the correlation coefficient 0.51. Thus, they are indeed, albeit modestly, correlated.

The highest peak of the Δ*Q*_*i*_ profile in Fig 5A is located at 166-th residue, which corresponds to the situation that the N-domain (1–143 residues) is mostly released from the ribosome exit tunnel. (Remember that the average number of residues in the exit tunnel is 28 as in S1 Fig). However, the translation profile in Fig 5B does not indicate any attenuation in this region. It seems that misfolding in the N-domain is not very probable in any CTFs and thus translational attenuation at this point is not required for successful folding.

### Conclusions

Comprehensively performing molecular simulations of co-translational folding (CTF) and refolding of SufI, we elucidated mechanisms of how translational attenuation can facilitate correct folding from structural perspectives. First, coarse-grained simulations showed that the codon-based CTF, CTF_codon_, exhibited higher probability of correct folding than the refolding did. When the translational attenuation is removed, the CTF_fast_ simulations resulted in the success rate similar to that by the refolding scheme. When the elongation was uniformly slowed down, the CTF_slow_ simulation gave essentially the same results as those of CTF_codon_. These are all consistent with recent experiments. On top, the simulations provided much of structural and mechanistic insights. Specifically for SufI, we found that the M-domain is least stable and can fold only when it is supported by the pre-folded N-domain. Once a segment of the M-domain is entangled with either N- or C-domain, an escape from the trap was difficult. Combining molecular simulations with biochemical experiments provided detailed mechanistic understanding of CTFs.

A recent theoretical study suggested that, under certain situations, fast translation can coordinate folding to the native structure [44]. Apparently, this is not the case in our SufI simulations. Whether slower or faster translation facilitates the correct folding depends on the folding kinetic network as was shown in [44]. We need some more investigations for specific proteins, through which we know which scenarios are more common.

We note that the current CG modeling has some limitations. One of the major limitations is on the time scales. Using the CG modeling, one cannot easily estimate the absolute time scales of folding and translation. Using a low viscosity in Langevin dynamics and structure-based potentials, we speeded up the folding kinetics some orders of magnitude. Translation kinetic parameters in the normal and slowed phases are not accurately known. This makes quantitative comparison difficult. Another limitation is the balance between the structure-based potential and the sequence-dependent terms, which was determined empirically here. Accurate modeling of these balances is highly desired in future work.

## Materials and Methods

### Model protein

In this study, we studied folding of a three-domain protein SufI [45] (Fig 1, PDB code: 2UXT). Starting with the PDB structure 2UXT, we removed the His-tag and modeled missing residues by MODELLER [46], resulting in the 443-residue long protein model. The model structured was refined by the energy minimization with AMBER [47]. Using Pfam’s [48], we defined three domains; N-terminal domain as 1–143, the middle (M-) domain as 160–300, and the C-terminal domain as region 314–443. Segments between two domains are termed linkers. The linker between M- and C- domains are rather long and extended.

### Coarse-grained model

In the simulation, one residue is represented by one CG particle which locates at Cα position. We used our in-house developing software CafeMol for all the simulations [49].

The potential energy function consists of the native-based AICG2+ potential (*V*_*Go*_) and non-local many body hydrophobic interaction potential (*V*_*HP*_). The total energy *V*_*total*_ for the refolding simulation is given as

$$V_{total}=aV_{Go}+bV_{HP}$$

where a and b are coefficients to control the balance between two terms. The potential for the CTF simulations is written as

$$V_{total}=aV_{Go}+bV_{HP}+V_{tunnel}$$

The native-based potential *V*_*Go*_ is defined as [32]:

$$\begin{array}{l} V_{Go}=\sum k_{b}{\left(b_{i}-b_{0i}\right)}^{2}+\sum V_{\theta i}(\theta_{i})+\sum V_{\varphi ,i}(\varphi_{i})\\ +\sum \varepsilon_{loc,i,i+2}\;\text{exp}\left[-{\left(r_{i,i+2}-r_{0,i,i+2}\right)}^{2}/2w_{i,i+2}^{2}\right]+\sum \varepsilon_{loc,i,i+3}\;\text{exp}\left[-{\left(\varphi_{i,i+3}-\varphi_{0,i,i+3}\right)}^{2}/2w_{i,i+3}^{2}\right]\\ +\sum_{i>j+3}^{native}\varepsilon_{non-loc,i,j}\left[5{\left(\frac{r_{0,ij}}{r_{ij}}\right)}^{12}-6{\left(\frac{r_{0,ij}}{r_{ij}}\right)}^{10}\right]+\sum_{i>j+3}^{non-native}\varepsilon_{ex}{\left(\frac{d}{r_{ij}}\right)}^{12} \end{array}$$

The first term keeps virtual bonds between consecutive amino acids, the second and the third terms represent statistical potential for virtual bond-angles and virtual dihedral-angles [50]. The fourth and the fifth terms define native-based local interactions [32]. The sixth term is non-local contact interaction for natively contacting pairs. The last term is a generic excluded volume interaction (See [32] for more details).

For the hydrophobic interaction, we take the function developed in[40], which is written in the form:

$$V_{\text{HP}}=-\sum_{i\in C_{\alpha }}\varepsilon_{\text{A}(\text{i})}^{\text{HP}}S_{\text{HP}}\;\left(\rho_{i}\right)$$

where $\varepsilon_{\text{A}(\text{i})}^{\text{HP}}$ is a parameter that reflects the hydrophobicity of amino acids for the amino acid type *A*(*i*). *S*_*HP*_ represents the buried-ness of the amino acid *i* and is defined as:

$$S_{\text{HP}}(\rho )=\left\{ \begin{array}{ll} \;1 & \;\rho >1\\ c_{\text{linear}}\rho +0.5(1-c_{\text{linear}})\left[1+\text{cos}\frac{\pi (1-\rho )}{1-\rho_{\text{min}}}\right] & \;\rho_{\text{min}}<\rho \leq 1\\ c_{\text{linear}}\rho & \;\rho <\rho_{\text{min}} \end{array}\right.$$

where *c*_*linear*_ and *ρ*_min_ are constants and *ρ*_*i*_ represents local density and is calculated by:

$$\rho_{i}=\frac{\sum_{j\in C\alpha ,j\neq i}n_{A(j)}u_{\text{HP}}(r_{ij},r_{\text{min},A(i),A(j)},r_{\text{max},A(i),A(j)})}{n_{\text{max},A(i)}}$$

where *n*_*A*(*i*)_ is the number of heavy atoms that defines the amino acid *A*(*i*) represents and *n*_max,*A*(*i*)_ is the maximum coordination number for particle type *A*(*i*). The function *u*_*HP*_ represents the degree of the contact between particle *i* and particle *j* and is defined as below a sigmoidal function:

$$u_{\text{HP}}(r,r_{\text{min}},r_{\text{max}})=\left\{ \begin{array}{ll} \;1 & \;r<r_{\text{min}}\\ \frac{1}{2}\left(1+\text{cos}\;\pi \frac{r-r_{\text{min}}}{r_{\text{max}}^{}-r_{\text{min}}}\right) & \;r_{\text{min}}<r<r_{\text{max}}\\ \;0 & \;r>r_{\text{max}} \end{array}\right.$$

We note that the described hydrophobic interaction potential was first developed for a CG model that uses different resolution from the current work. Thus we need to re-parameterize the function. We estimated parameters *r*_min,*A*(*i*),*A*(*j*)_ *r*_max,*A*(*i*),*A*(*j*)_, $\varepsilon_{A(i)}^{HP}$, *n*_max,*A*(*i*)_, and *n*_*A*(*i*)_ for each amino acid types in the following way. Using Dunbrack’s culled PDB set [51], we analyzed radius distributions of twenty types of amino acids. For details, if a distance between heavy atoms of two amino acids is less than *R*_*vdW*,*i*_ + *R*_*vdW*,*j*_ + *R*_*vdW*,*H*2*O*_, where *R*_*vdW*,*i*_ is the van der Walls radius of the atom *i*, we defined the distance between **Cα**'s as an effective distance, obtaining a set of radial distribution of 20x20 amino acid combinations. Then, we defined 95% confidence coefficient of their histograms as *r*_max,*A*(*i*),*A*(*j*)_ and we set *r*_min,*A*(*i*),*A*(*j*)_ = *r*_max,*A*(*i*),*A*(*j*)_ −4. $\varepsilon_{A(i)}^{HP}$ is taken from hydrophobic indices of Fauchere & Pliska [52]. All these parameters are included in the latest CafeMol and publicly available.

In the total potential energy *V*_*total*_, *V*_*Go*_(*R*) is responsible to make a globally funnel like energy landscape, while *V*_*HP*_(*R*) makes the landscape modestly rugged via physicochemical interactions. Thus, the balance of the two potentials is of central importance in the simulation. Since it is not straightforward to decide the coefficients in ab initio manner, in this work, instead, we took an empirical approach. At the native structure of SufI, we first calculated the potential energy *V*_*Go*_(*R*_*nat*_). We assumed that this value is a reasonable energy at the native structure and fixed this value at the native structure. We then express it as a linear combination of *V*_*Go*_(*R*) and *V*_*HP*_(*R*). Formally, it can be written as

$$V_{total}(R)=aV_{Go}(R)+(1-a)\frac{V_{Go}(R_{nat})}{V_{HP}(R_{nat})}V_{HP}(R),$$

where a free parameter *a* was decided fully empirically. With several values of *a*, we performed some preliminary simulations by the refolding scheme, estimating the probability of the successful folding, of which the criterion is defined below. We ended up with *a* = 0.8, by which about 20% of runs could reach the native structure. The coefficient *b* = 2.13 was derived from this procedure.

To reproduce a steric effect of ribosome exit tunnel and surface, we added a pure repulsive wall-and-tunnel potential *V*_*tunnel*_ defined as:

$$V_{tunnel}={\sum_{i}\varepsilon_{ex}\left(\frac{C}{d_{i}}\right)}^{12}$$

where *d*_*i*_ is the distance between the particle i and the wall-and-tunnel. The default parameters in CafeMol were used for *ε*_*ex*_ and *C*. The radius and length of the tunnel were set as *r*_*T*_ = 15 Å, *l*_*T*_ = 90 Å, respectively.

We note that all the interaction potentials here are temperature independent. Since hydrophobic interactions are effective interaction that itself depends on temperature, one can include temperature dependence as in Chan et al for more accurate modeling [53].

### Langevin dynamics

Molecular dynamics was simulated by the Langevin equation at the constant temperature *T*,

$$m_{i}\frac{d^{2}r_{i}}{dt^{2}}=F_{i}-m_{i}\gamma v_{i}+\xi_{i}$$

where *γ*_*i*_ is a friction constant and *ξ*_*i*_ is a random force. This random force satisfies 〈*ξ*_*i*_(*t*)〉 = 0, and 〈*ξ*_*i*_(*t*)*ξ*_*j*_(*t*')〉 = 2*γ*(*kT* / *m*)*δ*(*t* – *t*')*δ*_*i*,*j*_. The stationary distribution generated by this Langevin equation is the Boltzmann distribution for a given temperature *T*. The force *F*_*i*_ is derived from partial differentiation of the potential energy function. For numerical integration, we used the scheme in [54,55]. In the simulation, *γ* is 0.02, and a finite time step Δ*t* = 0.1.

### Translation elongation scheme

In simulations that mimic CTF, we increased the chain length of the nascent polypeptide one by one residue and used a wall-and-tunnel potential that represents the rough geometry of the ribosome exit tunnel (Fig 1E). The C-terminal residue of the nascent chain was fixed to the base of the ribosome tunnel during the elongation and is released when the final residue was “synthesized”. We assumed that the time scale for the covalent bond formation (the synthesis) is much shorter than time scale to wait the cognate tRNA and that for folding, and thus the synthesis is treated as the instantaneous change in the chain length. We also ignored any mechanistic factors possibly involved in the synthesis. Simply, we shifted the nascent chain toward the exit direction and added one residue at the base of the tunnel at the one-step elongation.

In a scheme that mimics the CTF rate that depends on codon (CTF_codon_), we used the translation elongation profile derived from Spencer’s algorithm [43]. Spencer’s algorithm generates relative translation profile for each organism (Fig 1B), by distinguishing Watson-Crick interactions from non-Watson-Crick (wobble) interactions. We note that we took this relatively simple algorithm, although there can be other algorithms. The number of tRNA genes for every codon was referred from Genomic tRNA database (gtrnadb.ucsc.edu) [56]. The mechanistic detail of the translational attenuation is unknown at the moment, and so, when an elongation rate is under a threshold, we defined the codon as a rare codon and the elongation was attenuated for 10^6^steps per one residue. For other residues, we used the elongation rate as 10^4^steps per one residue. The scheme was termed the CTF_codon_ (See Fig 1C).

To test the effect of synonymous substitution that remove the translational attenuation, we set the CTF in which the elongation rate is fast and uniform. A protein is elongated at the rate of 10^4^steps per one residue. This is termed as the CTF_fast_.

To test the effect of lowering temperature, we set the CTF in which the elongation rate is uniform and is slow (CTF_slow_). The elongation speed is 3×10^5^ steps per one residue.

As a control, we also set up the refolding scheme. In this scheme, a wall-and-tunnel potential was not used and the full-length SufI was present from the beginning. The initial unfolded conformation was prepared by constant temperature simulation at a high temperature for 10^7^ time steps from the native state. This was sufficient to prepare a fully unfolded structure.

In all four schemes, we ran 100 trajectories, and each trajectory is simulated for 3×10^8^ time steps, including the time for translation in the cases of CTF schemes. The comparison of three elongation schemes is given in Fig 1D.

### Criteria for folding

To judge whether SufI is folded or not, we introduced multiple native-ness scores, i.e., *Q*-scores.

In general, the widely used *Q*-score is defined as the number of formed native contacts relative to that presents in the native structures. First, an amino acid pair *ij* is defined as the native contact when one atom, except hydrogen, in *i*-th residue is within 6.5Å from at least one atom in *j*-th residue in the native structure. For natively contacting pairs, we check the Cα-Cα distance in a snapshot of folding simulations. If it is within 1.2 times the corresponding distance at the native structure, we assign the contact is formed in the snapshot.

The *Q*-score can be defined either for the full-length protein *Q*_*total*_, or for any part of the protein, both of which were used in this work. *Q*_*total*_ is convenient to quantify an overall native-ness by one value. When *Q*_*total*_ is above 0.95, SufI takes native state with high probability (this is called as a generous criterion for the native state). During the analysis, however, we noticed that, for multi-domain proteins such as SufI, the completion of folding cannot easily be assessed by *Q*_*total*_ alone. For example, we found that individual domains are all correctly folded, while some domain-domain interfaces are not. Since the number of native contacts for the domain interface is much less than those within the domains, these structures often take *Q*_*total*_ values close to one. (Even worse is that these values can be within the thermal fluctuation range of the *Q*_*total*_ at the true native state.) To distinguish these misfolded structures, we need to check *Q*-scores for every domain-domain interfaces, separately. Specifically for SufI, we introduced *Q*-scores for individual domains (three in total) as well as *Q*-scores for domain-domain interfaces (three in total), leading to six *Q*-scores of parts. When all the six Q-scores of parts are above their thresholds, we stringently assigned the structure well-folded (a stringent criterion for the native state).

### Discretization of states by Q-scores of parts

Q-scores for N-, M-, and C-domains and for N-M, N-C, and M-C domain-domain interfaces are classified by four thresholds. We located those thresholds at the local minima of statistical weight distributions. Specifically, thresholds of N-domain’s *Q*-score is [0.50, 0.63, 0.88, 0.94], those of M-domain: [0.30, 0.64, 0.78, 0.90], C-domain: [0.39, 0.59, 0.90, 0.95], N-M interface: [0.10, 0.30, 0.64, 0.83], N-C interface: [0.19, 0.44, 0.76, 0.90], M-C interface: [0.31, 0.56, 0.81, 0.90].

### Drawing folding network

To draw a folding network, we used a physical model of network, which is called a spring-electrical model [57]. In this model, each node is represented as a mass point and possesses a positive charge. If two nodes linked each other, the pair of nodes has an elastic energy. We seek locations of nodes that minimize the total “energy” function. We obtained an optimized network structure by a simulated annealing.

To discretize structural conformations, we classified six Q-scores of parts and six N-scores of parts. Here, N-score represents degree of formed non-native contacts and was defined as the number of formed non-native contacts relatively to the maximal number of the same contacts. Based on the thresholds, we can assign conformations to one of 5^6^×3^6^ nodes and represent a trajectory by a polygonal line that transits from a node to another. For simplicity, we removed any loops. Here, a loop is a sequence of transitions that start from and return to one node.

## Supporting Information

S1 Table Q-scores of intra-domains and inter-domains for snapshots depicted in Fig 2. (DOCX)

S1 Fig Observed numbers of residues that resided in the ribosome tunnel upon the elongation. The average is about 28 residues.
(TIF)

S2 Fig Temperature dependence of SufI unfolding. Starting from a denatured state, we performed folding simulations for 10^8^ time steps. Temperatur is given in CafeMol unit. The sudden drop in average Q-score was found at the temperatur 440, which corresponds to *T*_F_*. Folding simulations were conducted at 360, which corresponds to 0.82 *T*_F_.
(TIF)

S3 Fig Histograms of Q_total_-score in the final conformations. In each folding scheme, the last 100 snapshots (corresponding to 10^5^ time steps) are used.
(TIF)

S4 Fig Folding time course of standalone domains of SufI in normal (A) and in logarithmic (B) scales. (C) The linear fitting is used to obtain folding times of individual domain. Blue, green, and red curves correspond to folding of N-, M-, and C-domains.
(TIF)

S5 Fig Protein folding networks drawn from CTF_fast_ and CTF_slow_ folding schemes. The meaning of symbols are identical to those in Fig 4.
(TIF)

S6 Fig Non-native contact maps of the representative misfolded structures. The upper right triangle part shows the probability map of non-native map formed in the last 100 snapshots (corresponding to 10^5^ time steps) in representative trajectories. The lower triangl part shows the native contact map obtained from the native structure. The (i),(ii) and (iii) are three representative misfolded structures corresponding to the same symbols in Fig 3B.
(TIF)
