# Supplementary material for: How Co-translational Folding of Multi-domain Protein Is Affected by Elongation Schedule: Molecular Simulations
Source: PLoS Comput Biol. 2015 Jul 9;11(7):e1004356. doi: 10.1371/journal.pcbi.1004356 (PMC4497635; doi:10.1371/journal.pcbi.1004356)
Supplement: S1 Table — (DOCX) [file pcbi.1004356.s001.docx]

**S1 Table:** Q-scores of intra-domains and inter-domains for snapshots depicted in Fig.2.

Table S1-A-1: Q-scores for the snapshot at 0 time step in Fig. 2A.

|  | N-domain | M-domain | C-domain |
| --- | --- | --- | --- |
| N-domain | 0.05 | 0.0 | 0.0 |
| M-domain |  | 0.04 | 0.0 |
| C-domain |  |  | 0.04 |

Table S1-A-2: Q-scores for the snapshot at 7,239,000 time step in Fig. 2A.

|  | N-domain | M-domain | C-domain |
| --- | --- | --- | --- |
| N-domain | 0.483 | 0.343 | 0.034 |
| M-domain |  | 0.420 | 0.054 |
| C-domain |  |  | 0.608 |

Table S1-A-3: Q-scores for the snapshot at 29,759,000 time step in Fig. 2A.

|  | N-domain | M-domain | C-domain |
| --- | --- | --- | --- |
| N-domain | 0.984 | 0.537 | 0.0 |
| M-domain |  | 0.460 | 0.0 |
| C-domain |  |  | 0.822 |

Table S1-A-4: Q-scores for the snapshot at 51,133,000 time step in Fig. 2A.

|  | N-domain | M-domain | C-domain |
| --- | --- | --- | --- |
| N-domain | 0.926 | 0.488 | 0.0 |
| M-domain |  | 0.544 | 0.0 |
| C-domain |  |  | 0.867 |

Table S1-B-1: Q-scores for the snapshot at 13,678,000 time step in Fig. 2B.

|  | N-domain | M-domain | C-domain |
| --- | --- | --- | --- |
| N-domain | 0.157 | 0.0 | 0.0 |
| M-domain |  | 0.0 | 0.0 |
| C-domain |  |  | 0.0 |

Table S1-B-2: Q-scores for the snapshot at 123,102,000 time step in Fig. 2B.

|  | N-domain | M-domain | C-domain |
| --- | --- | --- | --- |
| N-domain | 0.990 | 0.910 | 0.025 |
| M-domain |  | 0.958 | 0.048 |
| C-domain |  |  | 0.0 |

Table S1-B-3: Q-scores for the snapshot at 176,205,000 time step in Fig. 2B.

|  | N-domain | M-domain | C-domain |
| --- | --- | --- | --- |
| N-domain | 0.970 | 0.965 | 0.748 |
| M-domain |  | 0.910 | 0.673 |
| C-domain |  |  | 0.741 |

Table S1-B-4: Q-scores for the snapshot at 265,000,000 time step in Fig. 2B.

|  | N-domain | M-domain | C-domain |
| --- | --- | --- | --- |
| N-domain | 0.970 | 0.940 | 0.992 |
| M-domain |  | 0.937 | 0.946 |
| C-domain |  |  | 0.967 |
