# Supplementary figures and images for: How Co-translational Folding of Multi-domain Protein Is Affected by Elongation Schedule: Molecular Simulations
Source: PLoS Comput Biol. 2015 Jul 9;11(7):e1004356. doi: 10.1371/journal.pcbi.1004356 (PMC4497635; doi:10.1371/journal.pcbi.1004356)

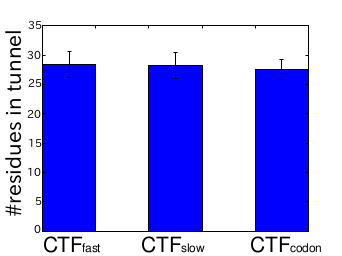

Supplement: S1 Fig — The average is about 28 residues. (TIF) [file pcbi.1004356.s002.tif]

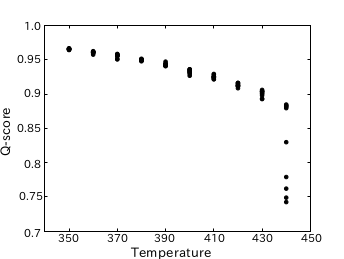

Supplement: S2 Fig — Starting from a denatured state, we performed folding simulations for 108 time steps. Temperatur is given in CafeMol unit. The sudden drop in average Q-score was found at the temperatur 440, which corresponds to T F*. Folding simulations were conducted at 360, which corresponds to 0.82 T F. (TIF) [file pcbi.1004356.s003.tif]

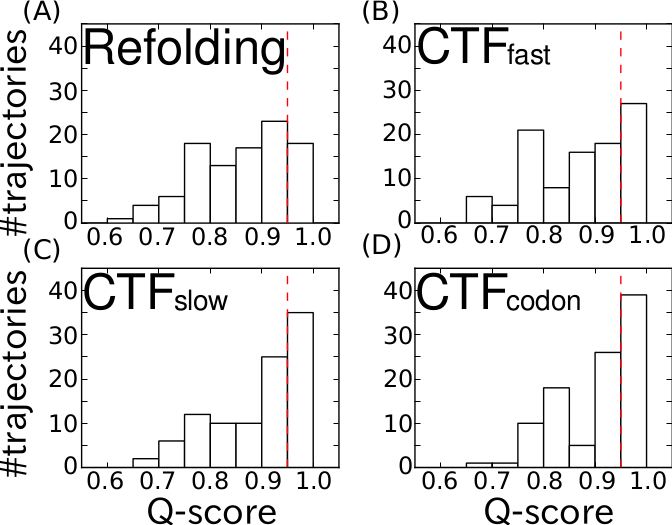

Supplement: S3 Fig — In each folding scheme, the last 100 snapshots (corresponding to 105 time steps) are used. (TIF) [file pcbi.1004356.s004.tif]

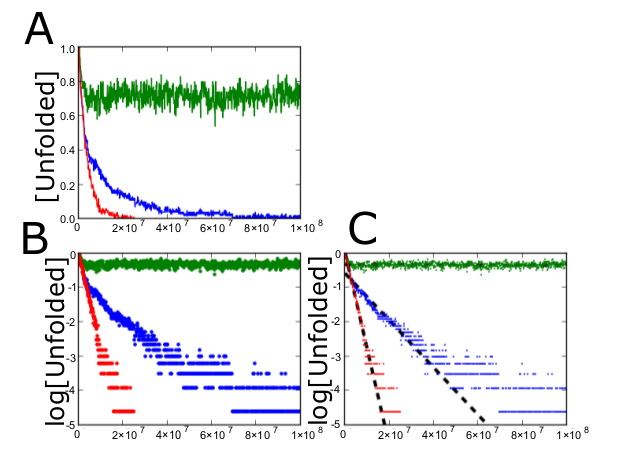

Supplement: S4 Fig — (C) The linear fitting is used to obtain folding times of individual domain. Blue, green, and red curves correspond to folding of N-, M-, and C-domains. (TIF) [file pcbi.1004356.s005.tif]

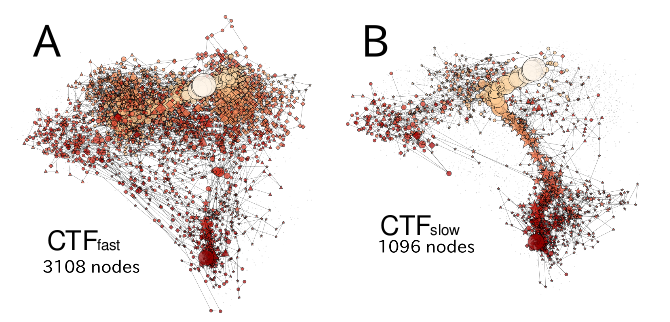

Supplement: S5 Fig — The meaning of symbols are identical to those in Fig 4. (TIF) [file pcbi.1004356.s006.tif]

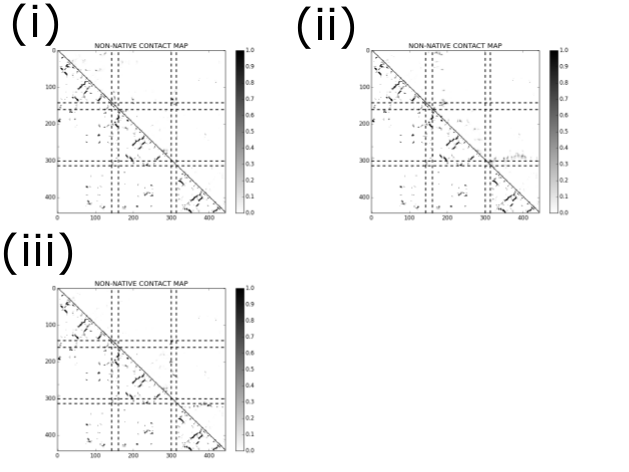

Supplement: S6 Fig — The upper right triangle part shows the probability map of non-native map formed in the last 100 snapshots (corresponding to 105 time steps) in representative trajectories. The lower triangl part shows the native contact map obtained from the native structure. The (i),(ii) and (iii) are three representative misfolded structures corresponding to the same symbols in Fig 3B. (TIF) [file pcbi.1004356.s007.tif]
